# Nanoscale strides: exploring innovative therapies for breast cancer treatment

**DOI:** 10.1039/d4ra02639j

**Published:** 2024-04-29

**Authors:** Sruthi Laakshmi Mugundhan, Mothilal Mohan

**Affiliations:** a Department of Pharmaceutics, SRM College of Pharmacy, SRM Institute of Science and Technology SRM Nagar Kattankulathur 603203 Tamil Nadu India mothilam@srmist.edu.in

## Abstract

Breast cancer (BC) is a predominant malignancy in women that constitutes approximately 30% of all cancer cases and has a mortality rate of 14% in recent years. The prevailing therapies include surgery, chemotherapy, and radiotherapy, each with its own limitations and challenges. Despite oral or intravenous administration, there are numerous barriers to accessing anti-BC agents before they reach the tumor site, including physical, physiological, and biophysical barriers. The complexity of BC pathogenesis, attributed to a combination of endogenous, chronic, intrinsic, extrinsic and genetic factors, further complicates its management. Due to the limitations of existing cancer treatment approaches, there is a need to explore novel, efficacious solutions. Nanodrug delivery has emerged as a promising avenue in cancer chemotherapy, aiming to enhance drug bioavailability while mitigating adverse effects. In contrast to conventional chemotherapy, cancer nanotechnology leverages improved permeability to achieve comprehensive disruption of cancer cells. This approach also presented superior pharmacokinetic profiles. The application of nanotechnology in cancer therapeutics includes nanotechnological tools, but a comprehensive review cannot cover all facets. Thus, this review concentrates specifically on BC treatment. The focus lies in the successful implementation of systematic nanotherapeutic strategies, demonstrating their superiority over conventional methods in delivering anti-BC agents. Nanotechnology-driven drug delivery holds immense potential in treating BC. By surmounting multiple barriers and capitalizing on improved permeability, nanodrug delivery has demonstrated enhanced efficacy and reduced adverse effects compared to conventional therapies. This review highlights the significance of systematic nanotherapy approaches, emphasizing the evolving landscape of BC management.

## Abstract 2

Nanotechnology revolutionizes breast cancer treatment by improving drug delivery, overcoming barriers, and reducing side effects. This review highlights its potential and superiority over conventional methods, transforming breast cancer management.

## Graphical abstract

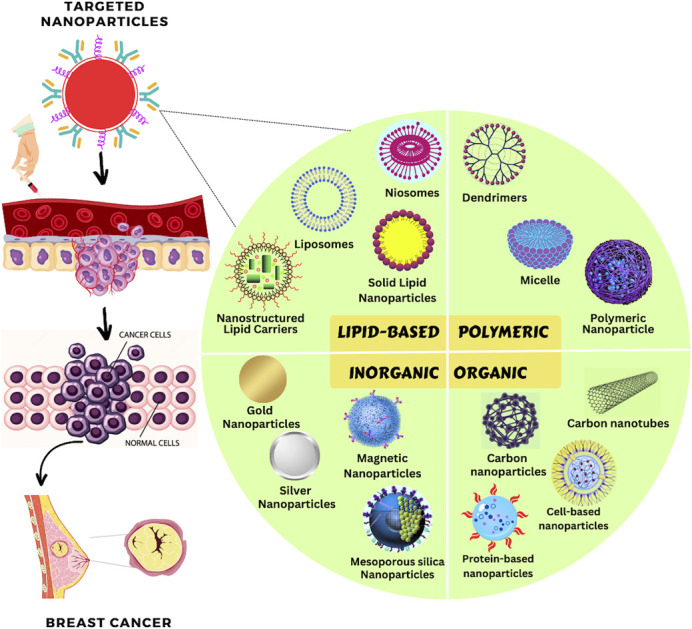

## 1. Introduction

Cancer can progress through the division of abnormal cells without ending or extending to other body tissues.^1^ Breast cancer (BC) is one of the predominant types of cancer in women, accounting for nearly 30% of all cancers, and has had a mortality rate of 14% in the last few years in India.^2,3^ BC is characterized by the expression of estrogen (ER), progesterone (PR) and human epidermal growth factor receptor-2 (HER-2).^4^ The receptors of BC cells determine the basis of targeted-treatment approaches and play a significant role in the prognosis and treatment of this disease. Like for other types of cancer, the standard therapy for BC includes surgery, chemotherapy and radiotherapy.^5^ Usually, anti-BC agents are administered *via* the oral or intravenous (i.v.) route, and the drug must pass through a number of barriers to reach the tumor site. These barriers encompass physical, physiological and biophysical factors.^6^ The objective of chemotherapy is to use cytotoxic anticancer drugs with or without surgery to compete with the division and development of tumor cells. Increased levels of certain proteins in tumor cells cause resistance to multidrug therapy, decreasing the effectiveness of chemotherapeutic agents. The pathology of BC is highly complicated among all cancers due to endogenous, chronic exposure to diverse intrinsic and extrinsic factors, as well as genetic interconnections.^7^ Considering these shortcomings in current approaches to cancer treatment, new beneficial solutions must be found. Delivering anticancer drugs to cancer tissues with the help of nondrug delivery in cancer chemotherapy increases bioavailability (in BAs) and greatly minimizes the adverse effects of drugs, which has recently become a research hotspot.^8^ Unlike standard chemotherapy, cancer nanotechnology disturbs cancer cells *via* improved permeability and leads to enhanced pharmacokinetic profiles in comparison with conventional therapy.^9,10^ NPs can provide tumor cells with large doses of therapeutic factors while bypassing normal cells.^11^ Nano-oncology attempts to remodel the delivery of chemical agents to targeted cancer cells through its merits: (i) it resolves problems with the drug's poor solubility and BA; (ii) it increases the permeability of therapeutic targets to tumor cells and allows for gradual drug release; and (iii) NPs are nontoxic, biodegradable, highly photoluminescent and small (1–100 nm) structures that can carry drugs.^12^ The application of nanotechnology in cancer treatment requires several nanotechnological tools, and in one review, it is unlikely that all of these tools will be included. This review will therefore focus solely on BC treatment, with emphasis on the successful use of systematic nanotherapy approaches over conventional methods for carrying BC anticancer agents.

## 2. Prevalence

Breast cancer affects women of all ages after puberty in every country on earth, although incidence rates tend to rise in later years. Despite substantial improvements in treatments for BC, over the span, death rates have remained relatively unchanged for approximately the last 3 decades.^13^ The incidence of BC increased by 130% between 2008 and 2020, from 1.38 million new cases to 1.67 million in 2012, 2.1 million in 2018, and 2.3 million new cases in 2020.^14^ In 2022, there were 2.3 million new cases diagnosed and 670 000 deaths worldwide due to breast cancer. Approximately upto 36% of oncological patients are breast cancer survivors.^15^ In the United States, there are presently over four million women who survived breast cancer. This includes women who have gone through the treatment as well as those who are currently undergoing it.^16^ An estimated 310 720 new cases of invasive breast cancer will be diagnosed in women in 2024, while there will be 2790 new cases in men. Additionally, there will be 56 500 cases of ductal carcinoma *in situ* (DCIS) in women in the US.^17^

Globally, there was a roughly two-fold variation in the overall cumulative risk of breast cancer before 40 years of age. Oceania had the highest risk (0.69%), followed by Europe (0.63%), the Americas (0.53%), Africa (0.49%), and Asia (0.38%). Based on national comparisons across 185 countries, South Korea had the greatest cumulative incidence rate (0.95%), followed by the United States, Canada, and the United Kingdom (0.77%, 0.61%, and 0.61%, respectively), and Guinea (0.13%) had the lowest.^18^ While the average incidence of breast cancer in Asia before 40 years of age is lowest, there is a six-fold variation across Asian nations.^19^ These discrepancies may be related to the growing “westernization” of lifestyle practices in some developing nations (*e.g.*, dietary modifications and reduced physical activity) that raise the risk of breast cancer, as well as the absence of public registries with reliable demographic data.^20^

**Fig. 1:**
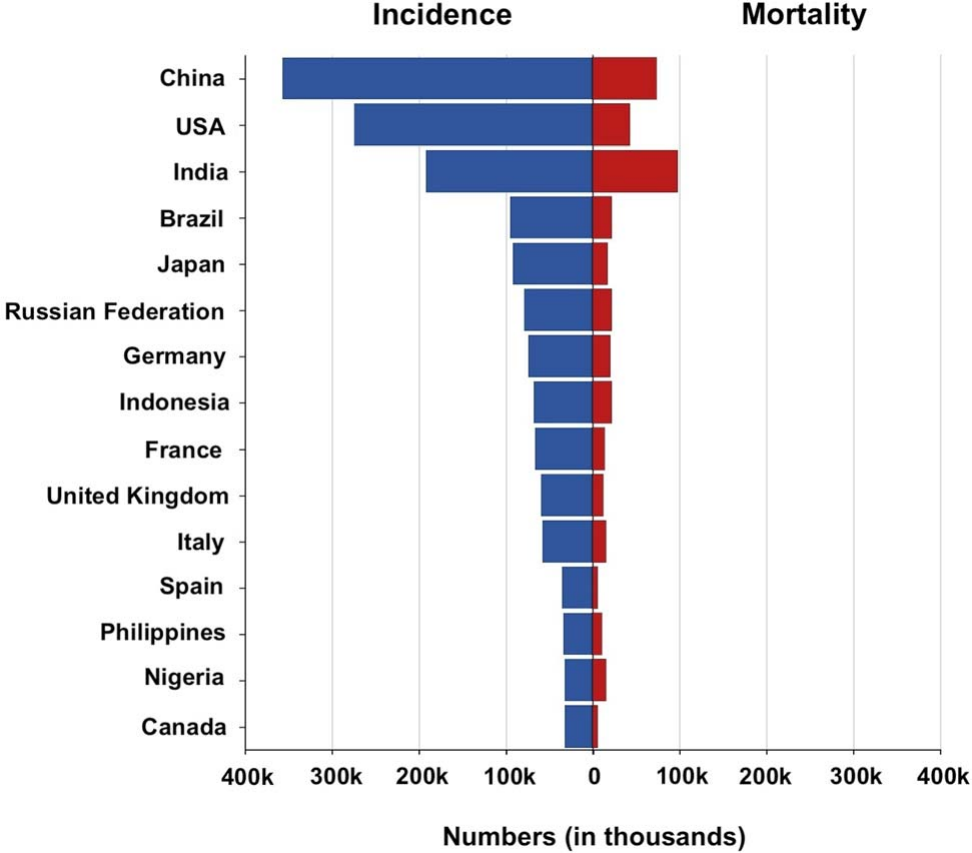
Absolute numbers of breast cancer incidence and mortality in various countries worldwide in 2022.

Based on the latest Globocan (WHO) data, Fig. 1 shows the absolute numbers of breast cancer incidence and mortality in various countries worldwide. In a study conducted by Yuyan *et al.*, in 2023, it was reported that there were 700 660 BC death cases worldwide in 2019 as compared to 380 910 incidents in 1990. Globally in 2050, there will be 1 503 694 death cases of BC (1 481 463 women and 22 231 males).^21^

**Fig. 2:**
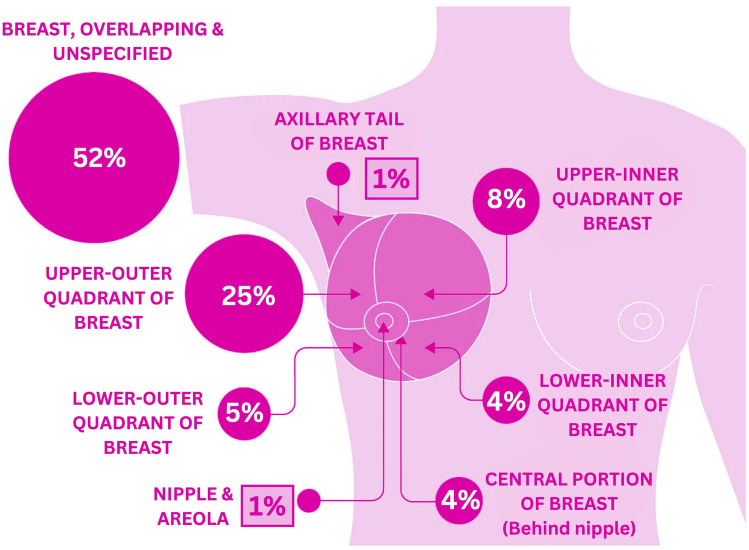
Percentage distribution of breast cancer incidence based on anatomical site in females (2016–2018, UK).

Additionally, Fig. 2 represents the distribution of BC cases diagnosed by anatomical site in females in the UK during 2016–2018.^22^

## 3. Etiology

The potential reasons for a hereditary link to BC include the following: (1) an elevated occurrence of BC in those with a family history of BC, (2) several members of the family affected with BC, and (3) cancer progression consistent with autosomal dominant inheritance. A predisposition gene for autosomal dominant cancer may be inherited and transmitted by both males and females.^23^ Drug resistance and the potential to metastasize to peripheral organs, such as the lymph nodes, lung, liver and bone, are responsible for the majority of BC deaths.^24^ A high birth weight is associated with an increased risk of BC, which might be due to changes in leptin, adiponectin, glucose, insulin, and insulin-like growth factor levels, as well as changes in the pregnancy estrogenic environment.^25^ Certain chemical contaminants, including 2,3,7,8-tetrachlorodibenzo-*p*-dioxin (TCDD), bisphenol, perfluorooctanesulfonic acid (PFOA), and benzo(*a*)pyrene, have recently been found to be associated with BC. Styrene, benzene, carbon tetrachloride, and formaldehyde are also substances that have been linked to an increased risk of BC.^26^

## 4. Clinical classification

Over the years, the categorization of BC has slowly progressed from merely being figurative based on morphological findings to being comburative, considering clinical characteristics, tissue-based biomarkers and genomes and involving protein expression profiles.^27^

**Fig. 3:**
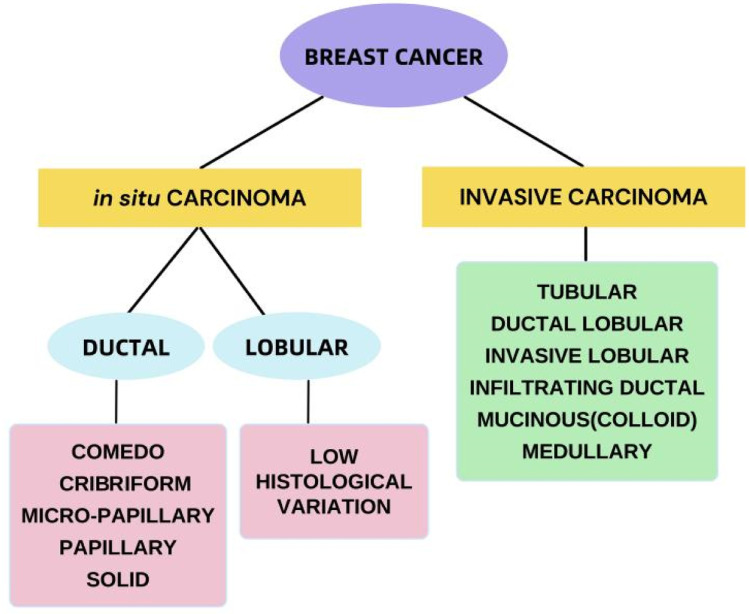
Histological classification of breast cancer.

Fig. 3 shows the histological classification of BC.^28^ The identification of biomarkers of cancer is among the most effective methods for identifying malignant and even premalignant lesions in the early stages.^29^ The study of gene expression arrays has contributed to the understanding of many markedly distinct BC subtypes.^30^ BC can currently be categorized as luminal A, luminal B, HER2-overexpressing, or triple-negative on the basis of genetic testing, histopathology and immunohistochemical analysis, based on the 2011 St. Gallen Consensus.^31^ As shown in Table 1,^32^ this classification involves the immunohistochemical analysis of ER and PR expression;, increased expression and/or induction of the HER2 oncogene;, and the use of the cell proliferation marker Ki-67 as a marker index to classify tumor subtypes. Most BCs overexpress the ER, although approximately 25% of BCs overexpress the HER. Nearly 15 percent of breast tumors do not express ER, PR or HER2; this type of tumor is known as triple-negative BC (TNBC) and is deemed the most problematic category of breast tumors.^33^

**Table 1:** Clinical classification of breast cancer subtypes

Intrinsic subtype	Clinical definition	Representation
Luminal A	✓ Expresses ER and/or PR	ER+, PR+ and HER2-
	✓ Does not express HER2	
Luminal B (2 categories)	✓ Expresses ER and/or PR	ER+, PR+ and HER2-
	✓ Does not express HER2-	
	✓ High Ki67 expression	
	✓ Expresses ER and/or PR	ER+, PR+ and HER2+
	✓ Also expresses HER2	
	✓ (Mostly in elderly)	
HER2-overexpression	✓ Does not express hormone receptors	ER-, PR- and HER2+
	✓ Expresses HER2	
Triple-negative	✓ Does not express ER, PR and HER2	ER-, PR- and HER2-

## 5. Nanodrug delivery for breast cancer treatment

All of these nanocarriers have the potential to provide enormous drug delivery potential and are therefore being explored for their potential use.^34,35^ By nature, nanomedicine can be applied clinically to materials with dimensions less than 100 nm, while devices with dimensions ranging from 100 to 200 nm also function as nanomedicines.^36^ Nanomedicine was reported to strongly decrease the peak free drug concentration (Cmax) while increasing the area under the curve (AUC) in plasma and tumor tissues. NPs have been shown to have an increased enhanced permeability and retention (EPR) effect.^37^

**Fig. 4:**
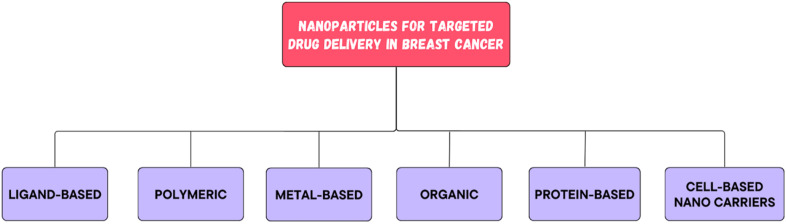
Types of nanoparticles used in breast cancer therapy.

**Fig. 5:**
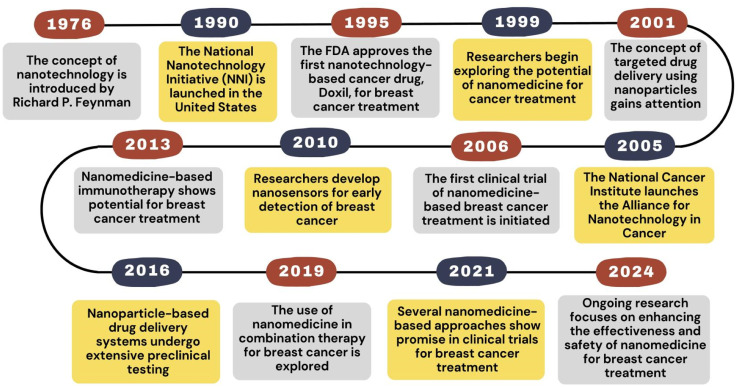
Historical timeline of nanomedicine for breast cancer treatment.

**Fig. 6:**
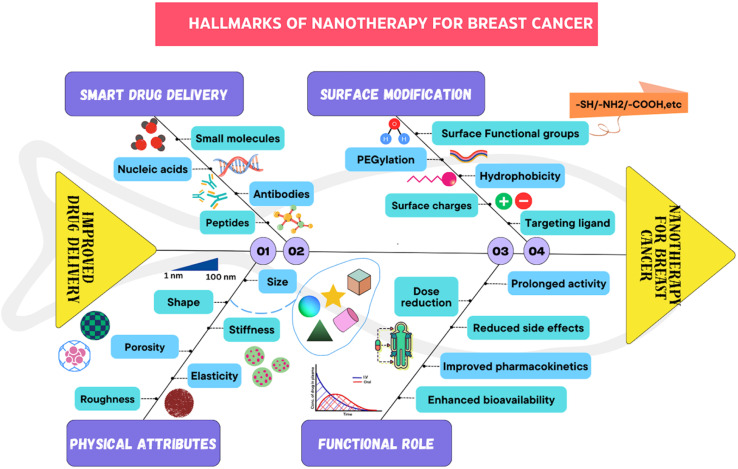
Hallmarks of nanotherapy in breast cancer.

Since nanomedicines are favorable for attaining the ‘right objective’ and ‘right exposure,’ they are meant to have an enhanced therapeutic index when compared to standard treatment.^38^ Highly permeable blood vessels in tumors are assumed to promote the deposition of nanoparticles in tumors.^39^ There are three possible routes for NPs to enter the interstitial tumor space through tumor blood vessels: through intercellular openings between endothelial cells, through transcellular holes and through endothelial cell fenestrae.^40^ However, the sizes of these pores differ among several tumor types, tumor microenvironments, and tumor species.^41^ The potential of altering the various attributes of NPs has transformed them into fruitful therapeutic vehicles for the treatment of cancer. For example, liposomes and polymer micelles enclose drugs inside a center, basically expanding their dissolvability, shielding them from deterioration, and inhibiting their untimely discharge into the circulation system.^42^ The nanoscopic size of NPs, deregulated vascular structure and upgraded EPR effects aid in preventing RESs, increasing the circulation half-life of drugs in the body and increasing the number of amassed NPs in tumor sites. The superficial presence of polyethylene glycol (PEG) on liposomes and different nanoparticles empowers a prolonged time course of the drug in blood vessels.^43^ Common types of nanomedicines that are effectively used in BC treatment are listed in Fig. 4. There are ample data on the use of nanotechnology in recent years to resolve the deficiency of chemotherapy in the targeted delivery of BC (Fig. 5). Using nanotechnology, the key features of breast tumors can be exploited to target drugs at the site. This basic strategy allows for increased specificity, boosting the success rate of anticancer chemotherapy. Fig. 6 illustrates the hallmarks of nanotherapy in the treatment of BC. In the last few decades, significant progress has been made in the development of potential nanomedicines for breast cancer treatment. Clinical application of nanomedicine in the treatment of breast cancer is still in the early stages of development, with only a limited number of products having been implemented. However, various approved products available on the market and a few clinical trials in progress are summarized in Tables 2 and 3.

**Table 2:** Approved nanoparticle-based formulations for breast cancer treatment^a^

Product	Company	Composition	Drug	Approval organization and year	Reference
Doxil®	Ortho Biotech	PEGylatyed STEALTH® liposomes composed of MPEG-DSPE, HSPC, CHO	Doxorubicin	FDA (1995)	61
				EMA (1996)	
Caelyx®	Schering-Plough	PEGylated liposomes composed of MPEG-DSPE, HSPC, CHO	Doxorubicin	EMA (1996)	62
Myocet®	Teva Pharmaceutical Industries Ltd	Liposomes (non-PEGylated) composed of PC, CHO, citric acid, and NaOH	Doxorubicin	EMA (2000)	63
Lipusu™	Luye Pharmaceutical Co. Ltd	Liposomes composed of lecithin and cholesterol	Paclitaxel	China (2003)	64
Abraxane®	Abraxis BioScience, Celgene Corporation	Colloidal suspension without solvent bound to albumin (active substance) in the form of a spherical nanoparticle	Paclitaxel	FDA (2005)	65
				EMA (2008)	
Genexol®-PM	Samyang Biopharmaceuticals	Lyophilized polymeric micellar formulation containing mPEG-PDLLA block copolymers	Paclitaxel	South Korea (2007)	66
Lipodox®	Sun Pharmaceutical Industries Ltd. (SPIL)	Liposomes with surface-bound mPEG	Doxorubicin	FDA (2013)	67
Kadcyla™	Genentech, Roche	Protein-based nanocompound containing ADC	Ado-trastuzumab emtansine	FDA and EMA (2013)	68
Pazenir®	Ratiopharm GmbH	Albumin-bound nanoparticles as powder for dispersion for infusion	Paclitaxel	EMA (2019)	69
Enhertu™	AstraZeneca/Daiichi Sankyo	Humanized monoclonal antibody trastuzumab covalently linked to the topoisomerase I inhibitor deruxtecan (a derivative of exatecan)	Trastuzumab deruxtecan	FDA, EMA, China (2019)	70
Trodelvy™	Immunomedics	ADC composed of active metabolite of irinotecan (SN-38) conjugated to a monoclonal antibody targeting trophoblast cell surface antigen 2, an epithelial cell surface antigen	Sacituzumab govitecan	FDA (2020)	71

a Abbreviations: PEG: polyethylene glycol; MPEG-DSPE: *N*-(carbonyl-methoxypolyethylene glycol 2000)-1,2-distearoyl-*sn*-glycero3-phosphoethanolamine sodium salt; HSPC: fully hydrogenated soy phosphatidylcholine; CHO: cholesterol; PC: phosphatidylcholine; NaOH: sodium hydroxide; mPEG: methoxypolyethylene glycol; PDLLA: poly(d,l-lactide); ADC: antibody-drug conjugate; EMA: European medicines agency; FDA: US food and drug administration.

**Table 3:** Current clinical trials in progress on nanomedicine for breast cancer^72–79^

Trial ID	Sponsors	Trial objective	Phases	Interventions	Type of nano-formulation	Study country	Recruitment status
NCT00629499	SCRI Development Innovations, LLC/Celgene Corporation	To evaluate the efficacy of nanoparticle albumin-bound (nab) paclitaxel/cyclophosphamide in early stage breast cancer(with trastuzumab in HER2-positive patients)	II	Nab paclitaxel, cyclophosphamide, transtuzumab	Nanoparticle albumin-bound	USA	Completed
NCT01644890	Nippon Kayaku Co., Ltd	To compare NK105 *vs.* paclitaxel in patients with metastatic or recurrent breast cancer	III	NK105	Micellar nanoparticle	Japan, Korea, Taiwan	Completed
NCT03671044	Jina Pharmaceu-ticals Inc	To evaluate the efficacy and safety of nanosomal docetaxel lipid suspension in triple negative breast cancer patients	III	Nanosomal docetaxel lipid suspension, Taxotere®	Nanosomal lipid suspension	USA, India	Recruiting
NCT04137653	Shengjing Hospital	To compare the therapeutic effect of nab-P with solvent-based paclitaxel in TNBC patients	III	Nab-paclitaxel, carboplatin	Nanoparticle albumin-bound	China	Recruiting
NCT04917900	West China Hospital	To evaluate the Pyrotinib in combination with albumin-bound paclitaxel and trastuzumab to neoadjuvant therapy efficacy and safety of Her2-positive early or locally advanced breast cancer	II	Pyrotinib combined with albumin-bound paclitaxel and trastuzumab	Nanoparticle albumin-bound	China	Recruiting
NCT06143553	Shanghai Yizhong Pharmaceutical Co., Ltd	To compare the clinical efficacy and safety of paclitaxel polymeric micelles for injection with TPC in HER2- metastatic breast cancer subjects	III	Paclitaxel, eribulin mesilate, capecitabine, gemcitabine HCl, vinorelbine tartrate, nab-paclitaxel	Polymeric micelles	China	Recruiting
NCT06199895	Liu Huang	To evaluate the efficacy and safety of paclitaxel polymeric micelles for injection for the treatment of patients with advanced breast and other cancer types	II	Paclitaxel polymeric micelles for injection	Polymeric micelles	China	Recruiting
NCT05949021	Mridula George, MD	To evaluate the efficacy, safety, and exploratory measures of liposomal doxorubicin and carboplatin combination therapy in the adjuvant setting for early stage triple negative breast cancer patients	II	Combination of liposomal doxorubicin	Liposome	USA	Recruiting

### 5.1. Lipid-based nanoparticles

Liposomal nanoparticles (LNPs) were first described in 1964 as spherical vesicle microparticles comprising single or multiple bilayered membrane structures with an aqueous center core.^44^ Lipophilic and hydrophilic medications may be incorporated due to the distinct bilayer compositions of the liposomes. Additionally, amphiphilic drugs, such as vincristine and DOX,^45^ may be encapsulated in the aqueous inner center of LNPs, which has been shown to reduce the cardiotoxicity of DOX compared to that of the unenclosed type.^46^ Considering the advantages of delivering amphiphilic drugs, it is important to note that the dimensions of LNPs are relatively larger (less than 50 nm), which can be overcome by coating LNPs with polymers. In liposomes, phospholipids are arranged in a bilayer pattern and thus result in a more soluble and stable drug formulation.^47^ This shows that encapsulating the drug effectively reduces toxicity due to the nontarget distribution of the drug. The biocompatibility of liposomes is optimal, and they are advantageous because of their good biodegradability, low immunogenicity, and low toxicity.^48^ Chemotherapeutic drugs in the form of LNPs were shown to be effective in clinical trials for treating BC with liposomal formulations, such as the Doxil liposome preparation.^49^ Doxil is the first anticancer nanomedicine used clinically and comprises a liposome encapsulating DOX. Doxil's PEGylated formulation reduces drug levels in blood without impacting the drug's anticancer activity because it enhances retention time and inhibits premature nanoformulation clearance.^50^ DOX is usually used for the successful treatment of Kaposi sarcoma and refractory breast and ovarian cancer (USFDA). Among several NPs used for cancer chemotherapy, LNPs are the first nanoparticle platform approved for systemic delivery of chemotherapeutics and for improved further use. There are a number of well-established LPN-based formulations, which include solid lipid nanoparticles (SLNs), nanostructured lipid carriers (NLCs) and many other types of liposomal formulations, which are discussed below.

#### 5.1.1. Solid lipid nanoparticles

SLNs are colloidal particles made of biodegradable, biocompatible and physiological lipids measuring approximately 50 to 1000 nm in size based on the preparation methods and composition; they are solid in nature at room and body temperatures and are safe for use^51^ SLNs are made of solid fat, surfactants and active pharmaceutical ingredients and are generated from natural or synthetic lipids.^12^ These NPs are used as an effective approach for improving the oral BA concentration of less aqueous soluble therapeutics due to their many advantages, such as ease of preparation, enhanced drug stability, increased drug content, efficient delivery of drugs and improved long-term stability.^52^ Compared with those of both MCF-7 and MCF-10A cells, SLNs harboring tamoxifen citrate and camptothecin exhibited improved efficacy.^53^ The evoked apoptotic mechanism in RES-loaded SLNs significantly represses on BC growth.^54^

#### 5.1.2. Nanostructured lipid carriers

Nanostructured lipid carriers (NLCs) are improved SLNs identified in the late 1990s; these NLCs contain a mixture of solid (fat) and liquid (oil) lipids that form a matrix.^55^ A few drawbacks of SLNs, including the ejection of the enclosed drug during storage and decreased loading of the therapeutic agent, could be effectively overcome by the addition of NLCs, which provide improved drug loading and minimal drug loss during storage.^56^ NLCs can be strongly assimilated by tumor cells and exhibit many favorable conditions, such as high drug encapsulation capacity, controlled drug delivery, improved drug stability and effortless extensive production.^57^ The poor oral BA content and solubility of quercetin were overcome by incorporating the drug into NLCs, the encapsulation efficiency was approximately 95%, and prolonged delivery of quercetin allowed significant cell death in MCF-7 and MDA-MB-231 cells. Additionally, drugs such as doxorubicin (DOX) and paclitaxel (PTX) were found to be effective against the MCF-7 and SK-OV3 cell lines and their multidrug resistant variants when enclosed in NLCs.^58^

#### 5.1.3. pH-sensitive liposomes

Due to their ischemic nature and pH of approximately 5 to 6.5, the unusually acidic tumor microenvironment (TME) of neoplastic tissues can be used to establish pH-responsive nanodrug delivery systems since the pH of healthy tissues and blood falls in the range of approximately 7 to 7.4.^59^ After the discovery of pH-sensitive drug delivery methods in 1980, pH-sensitive liposomal formulations were regarded as trustworthy methods for increasing drug availability at tumor sites (intracellular/extracellular). When used in conjunction with a ligand, this system can increase targeting and clinical effectiveness even more.^60^ These types of liposomes bear a negative charge and remain steady at neutral pH, but upon attaining acidic conditions at the tumor site, they exhibit pH-stimulated drug delivery *via* disruption of the lipid bilayer due to various molecular modifications in the lipid portion, such as protonation, deprotonation and decreased stability.^80^ pH-responsive liposomes of PTX showed rapid release of the drug at low pH, which was more effective in *in vitro* and *in vivo* BC models.^81^ As a result of pH variations, the ionization degree of pH-sensitive components, such as biopolymeric materials or phospolipids, changes due to their sensitivity to low pH, enabling them to accept or donate protons.^82^ A good example is phosphatidyl dioleoyl phosphoethanolamine (DOPE), which is a natural liposome that has a dual layered structure and a hexagonal structure at neutral and acidic pH values, respectively, leading to membrane rupture and loss of stability.^83^

#### 5.1.4. Thermosensitive liposomes

The use of thermosensitive liposomes (TSLs) is based on the concept that drug delivery can be facilitated by localized hyperthermia at the BC tumor site by combining the event of superheating with the merits of the liposomal structure. Traditional TSLs containing temperature-sensitive components and liposomes modified with temperature-sensitive polymers are two broad categories of TSLs.^84^ Extrinsic heat acts as a stimulus for drug delivery at the tumor site, which improves drug localization, reduces systemic side effects and offers integrated thermochemotherapy to tumor tissues.^85^ Docetaxel (DTX)-loaded liposomes significantly extinguished tumors at a rate 1.3-fold greater than that of tumors treated with drugs alone during *in vivo* animal studies of human BC in nude mice.^86^ The pioneering TSL formulation was developed in 1978 by Yatvin and others using DPPC and distearoyl phosphatidylcholine (DSPC) at a molar ratio of 3 : 1.^87^ Compared with those of nonthermosensitive liposomes, the growth of tumors from patients treated with cisplatin was markedly lessened, as observed in 2 individual tumor models (*i.e.*, MDA-MB-231 and MDA-MB-436).^88^

#### 5.1.5. Long circulatory liposomes

Generally, liposomes are prone to uptake by accelerated blood clearance and the reticuloendothelial system (RES) in the body after administration, and rapid opsonization results in easy elimination of liposomes. Liposomes have been investigated in a variety of ways to overcome their RES uptake and maintain them in circulation, including changing their size or altering their surface. To extend their circulation time, second-generation liposomes have a tweaked surface made up of glycoprotein, oligo- and polysaccharides, and synthetic polymers.^89^ Since the majority of liposomes accumulate in the liver and spleen and thereby might increase drug intoxication and side effects in these 2 organs, this issue can be efficiently mastered by formulating long-circulatory/circulating liposomes that are improved by water-soluble polymers such as PEG.^90^ Once long-term drug-charged circulating liposomes accumulate in cancer tissue, the drug can be liberated concurrently and regularly.^91^ Anticancer studies of BC cell lines (MDA-MB-231, MCF-7, and SKBR-3) revealed that treatment with liposomes containing glucoevatromonoside derivatives markedly decreased cell survival, and the formulations improved the targeting of these cancer cells.^92^

#### 5.1.6. Other liposomal nanodrug delivery systems

LPNs present various benefits when modified into self-micro/nanoemulsified drug delivery systems (SMEDDSs/SNEDDSs) and several other formulations other than the abovementioned liposomal preparations, including enhanced biodegradability, biocompatibility, and targeting of therapeutics.^93^ Payne, a British scholar in 1986, reported the existence of proliposomes for the first time to resolve difficulties such as aggregation, leakage of drugs and oxidative and hydrolytic degradation of phospholipids during storage. Proliposomes are formulated under pressure to minimize agitation, which results in a free-flowing powder by slowly mixing the drug with the liposomal material on the surface of a suitable carrier.^94^ It is known as a proliposome since the liposomal suspension has yet to be formed and is generated by the addition of water or an aqueous buffer prior to use. Based on the type of phospholipid incorporated in the preparation, proliposomes can be categorized into mixed micellar, liquid crystal and dry granular proliposomes. Curcumin, due to its anti-inflammatory, antioxidant and antitumor properties, has been reported to be use in BC and certain other cancers, yet its therapeutic application is limited owing to its low water solubility, instability, high metabolism and low abundance of BAs. Cationic PEGylated niosomes containing curcumin combined with PTX were produced by Alemi and coworkers to achieve an improved synergistic anticancer effects.^95^ Remarkably, many other studies have proposed that hybrid liposomes loaded with dipalmitoyl phosphatidylcholine (DPPC) and polyoxyethylene dodecyl ether can cause programmed cell death in various cancer cell lines, including human BC cells, lymphoma cells, and lung cancer cells.^96^ Immunoliposomes are another type of novel drug delivery tool that includes a combination of liposomes and targeted antibodies. Alteration of liposomes by monoclonal antibodies or their fragments results in immunoliposomes, which can selectively recognize and attach to antigens on the surface of the target site through immunoglobulins at the surface or their fragments, thereby facilitating drug accumulation at the targeted tumor site. These liposomes play a vital role in tumor targeting by penetrating cells either *via* endocytosis-derived fusion or by direct fusion with the plasma membrane, which decreases the aggregation of drugs in normal tissues, enhances drug efficacy and prevents drug toxicity.^97^ Compared with DOX-loaded liposomes with PEG-linked surfaces, anti-HER-2 DOX-loaded immunoliposomes are capable of improving the antitumor effects of drugs in xenograft models of BC.^98^ Third-generation targeted liposomes are prepared by changing to their surface by means of appropriate ligands and can be utilized for active and passive targeting of tumors. In passive targeting, they exhibit increased drug concentrations within the tumor owing to the EPR effect. Functionalized liposomes may carry the medication to a particular location, such as a cell organelle, because of specific receptor-selective associations with various forms of targeting ligands, such as peptides and antibodies.^99^ Hypoxia-responsive liposomes are primed by the injection of nitroimidazole derivative into the liposomal membrane. These patients exhibited a decrease in the hypoxic metabolism of the tumor and stimulated drug delivery due to liposomal degeneration. Such drug delivery results in oxygen-dependent delivery of the medication (DOX), which was verified by confocal laser scanning microscopy and near-infrared imaging. Liposomes sensitive to hypoxia showed particular cytotoxic effects on hypoxic tumor cells. These findings indicate that hypoxia-sensitive liposomes may be a successful path to cancer treatment.^100^ In addition to the above-described LPNs, photosensitive liposomes,^101^ magnetic liposomes,^102^ and ligand-modified liposomes^103^ include a few other liposomal drug delivery systems that are left undiscussed.

### 5.2. Polymeric nanoparticles

Polymer-based or polymeric NPs (PNPs) are made of natural or synthetic polymers and are self-assembled micelles approximately 100 nm in size. Owing to their enhanced durability, these NPs are extremely specialized in nature and exhibit positive rheological behavior. The release of chemotherapeutic agents is triggered after administration by degradation of the polymeric layer.^104^ The chemotherapeutic agent may be applied to the surface of PNPs through surface adsorption, chemical conjugation or encapsulation centered on the PNPs to target the tumor site.^105^ PNPs have the potential to charge active substances. In this way, the intracellular distribution of the active drug increases, and the drug is protected from degradation in a rigid matrix.^106^ In many trials, drugs entrapped against BC with polymeric micelles reportedly demonstrate greater efficacy than other drugs.^107^ Apart from using natural products, including cellulose and chitosan, as polymers,^108^ polymers synthetically prepared by nanoprecipitation, emulsification and salting-out methods are also employed for more specific biomedical applications of PNPs.^109^ PNPs of DOX HCl conjugated with hyaluronic acid and hydroxyethyl chitosan markedly increased the cellular uptake of the anticancer agents studied in HER2+ BC cell lines (MDA-MB- 453, MDA-MB- 435, and MCF-7).^110^ The various polymeric platforms used for anticancer nanotherapy include solid polymeric NPs, polymeric micelles, polymer conjugates, dendrimers, polymersomes, polyplexes, nanofibers and polymer–lipid hybrid systems.^111^ Owing to the improved stability offered by these NPs, the chances of RES accumulation and delay in biodegradation are marked as drawbacks. PNPs are efficiently modified to facilitate various benefits of targeting anticancer agents for BC by incorporating biodegradable polymers, immunotherapeutic agents and multiple chemotherapeutic agents.^109^

#### 5.2.1. Biodegradable polymeric nanoparticles

In nanocarrier platforms, biodegradable polymers are chosen due to their modified release properties, greater solubility and permeability, ability to release BAs, improved encapsulation ability and minimal toxicity. Chitosan, gelatin, polylactic acid (PLA), poly d,l-lactic-*co*-glycolic acid (PLGA), polycaprolactone, and polyalkyl-cyanoacrylate are widely used biodegradable polymers.^112^ A hydrophobic anticancer drug will be placed in the central hydrophobic bridging area inside the biodegradable PNP network, which undergoes surface modification through physical adsorption of hydrophilic polymer side chains. After accumulation within the tumor site, the NPs release the enclosed medication just after biodegradation and diffusion processes.^113^ pH-sensitive biodegradable polymers such as polyethylene oxide (PEO) become readily soluble at a pH below 6.5, enabling tumor-specific delivery of therapeutics such as PTX by encapsulating the drug and releasing it into the acidic breast TME.^114^ Biodegradable polymers offer extra benefit from oral chemotherapy, as patients may undergo chemotherapy immediately at home and have a better quality of life in the late cancer period. Oral chemotherapy frequently provides palliative care.^115^ In 2018, Farrokhi and his team constructed a DNAzyme that targets the expression of the BC cell oncogene c-Myc using a cyclodextrin nanocarrier. The formulation blocked the development of SMC cells and the cell line MCF-7 by 30–80%.^116^

#### 5.2.2. Polymeric micelles

Polymeric micelles are favorable supramolecular DDSs consisting of amphiphilic block copolymers with hydrophobic centers designed for aqueous insoluble drugs and hydrophilic layers loaded with molecules of hydrophilic drugs. Because of its semisolid hydrophobic center consisting of a biodegradable polymer, the polymer micelle measures a diameter of approximately 10–100 nm and is thus suitable for promoting many water-insoluble anticancer treatments.^102^ Genexol-PM, developed in Korea by Samyang Biopharm, is a micellar nanopolymer formulation of PTX indicated for the treatment of BC and non-small cell lung carcinoma (NSCLC) in South Korea.^117,118^ It comprises an amphiphilic diblock copolymer, monomethoxy poly(ethylene glycol)-block-poly(d,l-lactide) (mPEG-PDLLA) and PTX. Using an active targeting pathway, Baidya and his colleagues developed chrysin-charged folate-conjugated PF127-F68 mixed micelles to enhance oral BA and anticancer activity against human BC cells in 2019. The folate receptors in BC are activated by this combination. This technique was developed to investigate anticancer behavior in cell lines both *in vitro* and *in vivo*. The formulation revealed substantially higher Cmax and AUC0-t and improved anticancer action (5-fold) in an MTT assay (MCF-7).^119^ Furthermore, in a human BC model, BT474 xenograft mouse, bortezomib (BTZ)-loaded micelles exerted a better antitumor impact than free BTZ and suppressed BTZ *in vivo* hepatotoxicity.^120^

#### 5.2.3. Dendrimers

Dendrimers are the tiniest nanomaterials developed with dimensions of only 1.9 nm for G1 and 4.4 nm for G4, rendering their use simpler in some unique circumstances.^121^ Dendrimers are composed of polymeric star-shaped molecules that are continuously strongly branched and have a rigid 3D structure. They show 3 distinct sections, namely, a central core, branches and an external surface, with multiple surface operational groups.^122^ The most intensively researched group of materials is the polyamidoamine (PAMAM) dendrimer family, but the diversity of the building blocks is progressively increasing.^123^ Dendrimers bearing propylene imine (PPI) monomers are commonly employed in the delivery of drugs as well as nucleic acids in BC treatment, and they have also been equipped with ligands to target anti-BC drugs. Said and others synthesized a new water-soluble poly(propylene imine) dendrimer (PPI) modified with 4-sulfo-1,8-naphthalimid units (SNID) and its related structural monomer analog (SNIM) by simple synthesis.^124^ Gupta and others coupled 5th generation PPI dendrimers with FA to bind folate receptors and successfully achieved DOX delivery.^125^ These DOX-loaded FA-PPI dendrimers displayed faster release of DOX and greater cellular uptake by MCF-7 cells. Curcumin dendrimers (derived from *Curcuma longa*) have greater water solubility and have the potential to effectively induce apoptosis in BC cells.^126^ Thermosensitive branched OEG water soluble dendrimers could resolve the limitations of the currently used dendrimers. OEG dendrimers combined with gemcitabine (GEM) demonstrated beneficial tumor invasion and aggregation because of the longest peripheral PEG segments, resulting in marginally greater anticancer effects than those of GEM-linked PAMAM.^127^

#### 5.2.4. Multifunctional polymeric nanoparticles

Multifunctional PNPs are formulated for the simultaneous delivery of two or more anticancer agents, such as trastuzumab, incorporated with PTX NPs for targeted BC chemotherapy.^128^ These codelivery systems have several benefits, including delivering chemotherapeutic agents without harmful adjuvants, promoting synergistic effects, diminishing side effects of the drugs employed and achieving targeted delivery of cancer therapy^129^ Previously, a multifunctional dendrimer coupled with fluorescein isothiocyanate was formulated for scanning, FA as a tumor marker for targeting cancer cells that overexpress folate receptors and PTX as a chemotherapy medication.^130^

### 5.3. Inorganic nanoparticles

In addition to PNPS, metal-based NPs provide considerable versatility for drug distribution and diagnostics. Metallic NPs, including gold (Au NPs) and silver (Ag NPs), and magnetic NPs, including superparamagnetic iron oxide NPs (SPIONs) and quantum dots (QDs), play a helpful and effective role in cancer treatment owing to their low toxicity, fair size-to-volume ratio and outstanding thermal stability. These features have lead to improved targeting, silencing of genes, drug release and analytical assays for diagnosis. Almost all metallic NPs have the same framework, which consists of an electronic, magnetic and optical center and a shield that is primarily an organic surface coating.^131^ Additionally, metallic NPSs could be used for integrating optical and magnetic imaging in conjunction with fluorescent active molecules.^132^

#### 5.3.1. Gold and silver nanoparticles

Gold NPs were first identified by Michael Faraday.^133^ These NPs have dimensions of approximately 130 nm and are stable, with good biocompatibility and no toxicity.^134^ Herceptin®, a humanized anti-human HER-2 antibody conjugated with PAMAM dendrimers consisting of Au NPs and gadolinium (Gd), may be utilized for the early diagnosis and therapy of human HER2+ BC.^135^ Sun *et al.* engineered DOX-loaded Au NPs that are capable of transporting the medication efficiently to BCSCs and substantially enhancing antitumor efficiency.^136^ Ghosh *et al.* documented the use of Gloriosa superba tuber extract to prepare gold and silver nanoparticles and reported that AgNPs and AuNPs have excellent anticancer effects on MCF-7 cells.^137^ An American study by Nima and coworkers recently described the ability of nanotherapeutics (DOX) to treat BC and prostate cancer cells utilizing silver-decorated gold nanorods.^138^ Hibiscus rosasinensis petal extracts and chitosan were used to produce antioxidants such as vitamin C- and vitamin E-encapsulated NPs and Ag NPs. Chitosan polymers can bind to each other and are biocompatible, biodegradable and cationic in nature. These NPs were proven to be significantly hemocompatible and had a strong encapsulation performance of almost 76%. In fact, slightly greater antitumor behavior toward BC cell lines (MCF-7) was observed.^139^ Gold NPs can be directed toward BC cell mitochondria and may be successful at triggering apoptotic cell death and activating tumor cell damage, which may be useful in BC photothermal therapy.^140^

#### 5.3.2. Magnetic nanoparticles and SPIONs

Magnetic NPs can be generated by means of a magnetic field. Magnetic materials such as nickel, cobalt and iron are used to construct these NPs.^141^ These NPs are being utilized in MRI with the advancement of nanotechnology, owing to their ability to bind to the target site and thus support drug release technology.^142^ SPIONs are approximately 1 to 100 nm in size and are composed of an inner magnetic core made of magnetite (Fe_3_O_4_) or maghemite (γ-Fe_2_O_3_). Compared with magnetite, the latter is the most suitable because of its minimal chance of causing Fe(iii) toxicity in the body.^143^ The magnetic core is often shielded by a water-soluble stabilizing layer such as polymers that enables targeted biomolecules to be delivered to the target site. The most widely used biopolymers for stabilization include polysaccharides, PEG, dextran, alginate and polyacrylic acid (PAA).^144^ SPIONs are concurrently employed in tissue regeneration, immunoassays, magnetic field cell imaging and drug delivery.^145^ In 2018, Nosrati and others generated methotrexate-conjugated l-lysine-coated Fe_2_O_3_ magnetic NPs for BC treatment. This research was performed on BC cells from MCF-7 cells. The preparation had a negative zeta potential and a particle size < 100 nm, indicating major anticancer effects.^146^ Poller *et al.* further contrasted the influence of 3 separate forms of SPIONs on BC cells, which differed in scale, form, zeta potential and surface coating. Comparisons of the cellular absorption, magnetic properties and cytotoxic effects of the SPIONs are shown. Among the three kinds of SPIONs involved in BC therapy, namely, dextran-coated (SPIONDEX), lauric acid-coated (SPIONLA) and SPIONLA, which include human serum albumin (SPIONLA-HSA), the SPIONLA demonstrated the maximum cell absorption and cell cytotoxicity against BC cells.^147^

#### 5.3.3. Quantum dots

Quantum dots (QDs) play a vital role in BC therapy due to their significant optical properties. QDs are fluorescent NPs with a size of approximately 2 to 10 nm that are composed of a core of hundreds to thousands of atoms of elements belonging to groups II and VI (for instance, cadmium, technetium, zinc and selenide) or group III (Eg, tantalum) and V (Eg, indium).^148^ QDs serve as a powerful diagnostic tool for BC in addition to therapeutics due to their ability to emit narrow symmetrical peaks with minimal overlap between the spectra. QDs that are emitted at different wavelengths were studied in an analysis of the cell lines MCF-7 and BT-474, and it was observed that these two cell lines produce specific amounts of the following 5 biomarkers: ER, PR, EGFR, mTOR and HER2. The QDs were therefore paired with the main antibodies of these protein biomarkers and used for identification by simultaneous quantitative and multicolor detection.^149^ Nevertheless, *in vivo*, the use of QDs in imaging and treatment is restricted by the toxic impact of heavy metal cores.^150^ Additionally, they require coating surfaces with polymers or multilayer ligand shells to provide considerable water solubility owing to their hydrophobicity. The QDs of the somatostatin analog veldoreotide were used to biomap and reduce tumor development in patients with BC and are currently in a phase I clinical trial.^151^

#### 5.3.4. Mesoporous silica nanoparticles

Mesoporous silica NPs (MSNs) have attracted enormous interest in the field of nanotherapeutic drug delivery because of their unique physicochemical properties.^152^ Additionally, MSNs possess a large surface area and pore volume and can possibly alter the pore size in addition to having an easily modifiable surface, enabling them to act as effective tools for drug delivery and imaging.^153^ NPs bearing either medications or genetic material are contained inside the pores of MSNs, whose shape and scale are designed to reduce RES sequestration and improve tumor absorption by increasing vessel width, strong adhesion and cell assimilation.^154^ A study published by Tsai and others revealed the incorporation of an anti-HER2/neu monoclonal antibody in the form of NPs utilizing green fluorescent MSNs as a DDS to selectively target BC cells.^155^ Wu *et al.* produced Bcl-2 siRNA-packed mesoporous polymer nanospheres in 2019 to bind to the BC FA receptor. The NPs were administered competently to BC cells and strongly inhibited the expression of the Bcl-2 mRNA, which is involved in BC cell death.^156^ Conversely, one significant downside of MSNs is that they lack the capacity to penetrate into the tumor mass.^157^

### 5.4. Organic nanoparticles

Organic NPs have been widely utilized and studied for various oncologic purposes in recent years for diagnosis and cancer therapy. Several researchers have explored hybrid organic nanocarriers for drug delivery and scanning and have reported successful decreases in tumor size, efficient concentrations of drugs at active sites and influential diagnoses of cancers.

#### 5.4.1. Carbon-based nanoparticles

Carbon nanomaterials such as fullerene, graphene, carbon nanotubes and carbon dots (CDs) have attracted increased interest in BC therapy because of their special physicochemical, optical and biological properties.^158^ Fullerenes, carbon nanotubes (CNTs) and graphenes may be fabricated to detect and manage cancer on the surface with imaging tools and drugs, and such carbon-derived NPs possess various desirable qualities, such as a compact scale, large specific surface region, robust useable surface groups, favorable biocompatibility, low toxicity, and distinct optical and thermal characteristics.^159^

##### 5.4.1.1. Carbon nanotubes

CNTs are fullerene allotropes with thin, long, cylindrical, branched, hollow-framed hydrophobic carbon atom constructs that are insoluble in water and other organic solvents, and their toxicity in a biological medium is the main limiting factor. CNTs possess walls made of layers of graphene rolled at a precise angle and are known as single-walled (SWNT) or multiwalled (MWNT) nanotubes based on single or multiple graphene sheets.^26^ The amine-functional fullerene nanostructure has outstanding fluorescence properties and has the potential to penetrate *in vitro* BC cells easily (MCF-7). Fiorillo reported that breast cancer stem cells (BCSCs) are prone to carbon nanotube therapy based on graphene oxide and have good potential to target BCSCs.^160^ Hence, CNTs are successful transporters for drug distribution. However, the processing of CNTs cannot be performed easily. CNTs also have some disadvantages, such as solubility and biodegradability.^161^

##### 5.4.1.2. Carbon dots

CDs were introduced in 2004 as a type of carbon-based NP, and in the early period of their development, major studies focused on photoluminescence (PL) by means of several synthetic methods, starting materials and surface modifications.^162^ The application of CDs in BC treatment occurred for the first time in 2013. Hsu *et al.* published a study of green tea-derived CDs in 2013, which highlighted cancer cell suppression activity^163^ using the cancer cell lines MCF-7, MDA-MB-231, and HeLa (human cervical carcinoma) cells. As the accumulation of CDs increased, the percentage cell viability decreased to approximately 20, 18, and 68%, respectively, which was attributed to H_2_O_2_ and reactive oxygen species (ROS) generation and reflected the significant suppression of BC cell lines. Further research on BC therapy was subsequently conducted in 2018 by Kong *et al.* by combining CDs with DOX through electrostatic interactions, and the conjugates generated greater cell absorption and anticancer effects on MCF-7 cells than did free DOX.^164^

#### 5.4.2. Protein-based (viral) nanoparticles

The use of protein-based NPs is another promising flexible approach for targeted medication delivery. Viral NPs are those corresponding to the protein envelopes or capsids of viruses that are biocompatible and biodegradable, which makes them suitable drug carriers.^165^ The viral NPs derived from plant viruses and bacteriophages are particularly effective because they exhibit low risks of pathogenicity and undesired side effects in the human body.^166^ Nonetheless, viral NPs are noninfectious because they lack a viral genome. Potato virus X (PVX) was bonded and propagated to athymic mice harboring human MDA-MB-231 BC xenografts by Le *et al.* in 2017, and PVX-DOX treatment reduced tumor development.^167^ Thus, the use of PVX, a form of viral NP that originates from plants, paves the way for malignant therapy using viral NPs. In addition to PVX, influenza viral NPs altered by the securing of glycophosphatidylinositol (GPI) and the HER2 antigen by protein transfer likewise resulted in increased defense against HER2-expressing tumor cell proliferation in a murine BC model, and updated GPI-HER2 influenza viral NPs were added to the development of HER2-specific IgG and improved HER2-specific Th1-type immunity compared with GPI-HER2 vaccination alone.^168^

### 5.5. Cell-based nanocarriers

The bulk of the cells used in these systems have been established to have potential in tumor areas, such as the center of tumor hypoxia, so they are generally welcomed. The use of host cells as NP-releasing vehicles to address challenges in anticancer drug targeting for BC is thought to be a very useful method. Monocytes were stocked *in vitro* with gold nanoshells and then leached onto spheroids of BC tumors with nanoshell-coated macrophages. Furthermore, these experiments showed the use of macrophages for targeted phototherapy focused on the spheroid design of BC tumors.^169^ Although conventional DDSs, such as liposomes and polymeric NPs, have been used as carriers of anticancer and antifungal agents in recent years, certain negative features, such as biocompatibility and long-term safety issues, prevail.^170^ These limitations can be efficiently solved by the use of exosome-based drug delivery systems, which exhibit great physiological stability. Since exosomes have a hydrophobic nucleus, they promote the storage of water-soluble drugs. There are three distinct groups of extracellular vesicles, namely, microvesicles (MVs), ranging from 100 to 1000 nm in diameter; apoptotic bodies, ranging from 1 to 5 μm in diameter; and exosomes, ranging from 30 to 100 nm.^3^ Exosomes are efficient transporters of large molecules such as proteins. For instance, surface modifications develop with exosomes produced from immature dendritic cells in mice, and these cells are modified to establish the Lamp2b exosomal membrane protein, which is fused with the iRGD peptide unique to ac integrin.^171^ The electroporation-isolated exosomes were assembled with DOX, and the efficiency of encapsulation was estimated to be 20%. These engineered exosomes were intravenously administered to rats, after which DOX entered the tumor, and as a result, tumor development was inhibited.^172^ DOX-treated exosomes were used to treat BC and TNBC for successful targeting and delivery to αv integrin-positive BC cells and thereby showed a reduction in immunogenicity and toxicity.^173^

## 6. Targeting potential of nanoformulations for breast cancer treatment

Nanotechnology nanoformulations show significant promise for transforming the treatment of breast cancer since they can precisely target cancer cells while limiting toxicity and off-target effects. Improved pharmacokinetics, increased solubility of poorly water-soluble drugs, extended circulation time, and the possibility of targeted drug delivery to tumor sites through passive and active mechanisms are just a few benefits these nanoscale drug delivery systems have over traditional therapies.^174^

One of the key advantages of nanoformulations is their ability to passively target cancerous tissues through the enhanced permeability and retention (EPR) effect. This phenomenon takes advantage of the leaky vasculature and poor lymphatic drainage commonly found in tumors, allowing nanoparticles to preferentially accumulate within the tumor microenvironment.^175^ By encapsulating chemotherapeutic agents within nanoparticles, drug concentrations at the tumor site can be increased while reducing systemic exposure and associated side effects. Additionally, surface modification of nanoparticles with targeting ligands such as antibodies or peptides enables active targeting of specific molecular markers overexpressed on the surface of breast cancer cells. This targeted approach enhances the selectivity of drug delivery, further improving therapeutic efficacy while minimizing damage to healthy tissues.^176^

Zhang *et al.* 2023 developed PLGA shell PANP nanoparticles with perfluoropentane (PFP), Paclitaxel (PTX), and anti-miR-221 inhibitor and monitored them *in vivo* using ultrasound-triggered PFP vaporization. RAW-PANPs enriched in tumor tissues after injection into tumor-burden mice. Ultrasound cavitation explosion released PTX in the tumor. The release of anti-miR-221 improved tumor cell PTX sensitivity. Therefore, RAW-PANPs suppressed TNBC cell proliferation *in vitro* and tumor growth and progression *in vivo*. The treatments did not affect the heart, kidneys, or liver and the study developed a macrophage-carried, ultrasound-triggered, cancer cell-targeted chemotherapeutic system and a miRNA-based approach to improve cancer cell drug sensitivity to treat treatment-resistant TNBC patients. This novel study used macrophages to transport nanoparticles to tumors and ultrasonically break them to release miRNA and PTX.^177^ In another recent study by Li *et al.* 2023, autopilot biohybrids (Bif@BDC-NPs) which deliver doxorubicin to tumors were developed. The study involved preparation of albumin-encapsulated DOX nanoparticles (BD-NPs) coated with chitosan for breast cancer chemotherapy and anaerobic Bifidobacterium infantis (Bif) as self-propelled motors form this combination. Bif@BDC-NPs accurately attach hypoxic tumor tissue and promote drug accumulation at the tumor location, causing tumor cell death due to Bif's anaerobic characteristics. Therefore, Bif@BDC-NPs, a bacteria-driven oral drug delivery method, bypasses several physiological barriers and has significant potential for precision solid tumor treatment.^178^

Polydopamine NPs were formulated to deliver PTX and Trastuzumab to HER2+ breast tumors. PDA NPs, despite not being loaded with taxane or anti-HER2 antibody, have shown remarkable antitumor activity *in vitro* in HER2+ conventional cell cultures and breast tumor spheroids.^179^ Encapsulation of diosgenin in PLGA nanoparticles, coated with folic acid-chitosan, yielded a stable formulation with potent anticancer effects against TUBO breast cancer cells. *In vitro* and *in vivo* studies demonstrated dose-dependent inhibition of tumor growth and apoptosis induction.^180^

These studies highlight the potential of nanoformulations to enhance the efficacy of breast cancer treatment by improving targeting specificity, therapeutic outcomes, and diagnostic capabilities. Nanoformulations are as miniature missiles that target breast cancer either by becoming trapped in leaking tumor vessels, which increases the concentration of the substance at the site (passive targeting) or by modifying their surface, they are able to target cancer cells while causing minimal damage to healthy tissue (active targeting). This focused strategy provides promise regarding improved treatment results and diminished adverse effects. Continued research efforts in this field are critical to translating these promising nanoformulation strategies into clinically effective therapies for breast cancer patients, ultimately advancing personalized medicine and improving survival rates.

## 7. Challenges in the development of nanomedicine for BC treatment

Nanotechnology has great potential for breast cancer treatment, but many barriers must be addressed before its use in clinical practice.

Ensuring the success of nanopharmaceuticals is hampered by economic considerations. The production process and raw material costs are relatively high, which drives up the price of their products. For instance, the production of medications like Paclitaxel and Doxorubicin, which are free pharmaceuticals, is significantly less expensive than that of Abraxane™ and Doxil™^.^^181^ It is projected that the complete commercialization process for a novel nanodrug will take around 10–15 years and cost approximately $1 billion. Therefore, in order to justify their higher cost in comparison to traditional therapies, the clinical benefits of nanomedicines must be evident.^182^ Given that even minute changes in the manufacturing process can have a substantial impact on properties like size, shape, composition, drug loading and release, biocompatibility, toxicity, and *in vivo* outcome, the scalable and controlled manufacturing of nanomedicines under good manufacturing practices (GMP) conditions poses special challenges. As a result, several techniques should be used to characterize nanomedicine products batch by batch.^183^

Furthermore, it is crucial to address unique concerns regarding nanomedicines designed for human use, including sterility. Consequently, one of the greatest obstacles in the development of nanomedicines is to identify a method of sterilization that preserves the physicochemical properties and stability of the therapeutic molecules without compromising them. Particular care must be taken when developing nanomedicines utilizing biological molecules, such as proteins, because of their extreme vulnerability to degradation by sterilization methods.^184^ Particular attention must be paid to the endotoxin contamination in this context. Endotoxins can cause severe health problems, and contamination with endotoxins causes the failure of over thirty percent of nanoformulations during early preclinical development. Consequently, the endotoxin content of nanomedicines must be meticulously assessed utilizing suitable methodologies. Additionally, it is difficult to characterize the storage and stability characteristics (shelf life) of nanomedicine products. Additionally, the characteristics of nanomedicines can be modified through storage in lyophilized form or aqueous solutions.^185^

It is necessary to evaluate the toxicological effects of nanomaterials; however, certain toxic effects remain largely unknown. The derived toxicological data, with the exception of a few observations that have been reported thus far, are inconsistent and contradictory. The implementation of guidelines to standardize preclinical nanomedicine research is obligatory. Such guidelines would facilitate modeling, quantitative comparisons, reproducibility, and cost-effectiveness, thereby enhancing the utility, effectiveness, and safety of nanoformulations.^186^ Furthermore, they would aid in the rapid translation of basic research into clinical practice. Furthermore, regulatory concerns play a significant role in the advancement of technologies utilized for the characterization and quality monitoring of nanopharmaceuticals. Regulatory determinations concerning therapeutic nanomedicines are established through the subjective evaluation of risks and benefits by an individual; this tedious procedure can cause regulatory setbacks for nanomedicine products.^187^ Integrating the efforts of international consortia comprised of clinicians, academics, pharmaceutical companies, and regulatory authorities should be among these actions taken in order to improve the clinical efficacy and patient outcomes of these nanopharmaceuticals that target cancer.^188^ Numerous nanoformulations, ranging from inorganic to organic nanoparticles, with a variety of formulation and production techniques, great flexibility, and control over size and shape, have been studied in recent publications. They are loaded with active compounds and several chemotherapeutics, and they have been functionalized to enable targeted therapy.^198^ This inadequate rate of clinical translation, however, works in opposition to the positive outcomes of preclinical research. In fact, there are currently very few drugs based on nanotechnology that are approved for use in clinical trials. In order to enhance clinical trials, Van der Meel and colleagues proposed using smart patient stratification techniques in cancer nanomedicine. These techniques include imaging-based tumor accumulation to select trial candidates and probes and procedures to evaluate the tumor microenvironment.^199^

Furthermore, they demonstrated that the meticulous rational design of pharmacological combination regimens will enhance the pharmacokinetic and/or pharmacodynamic advantages and suggested the implementation of creative techniques for designing modular (pro)drugs and drug-delivery systems, in addition to library screening, in order to increase the likelihood of successful preclinical testing of formulations. Performance, translation, and exploitation of nanomedicine will all be enhanced by these smart strategies.^200^

## 8. Insights on personalized protein corona in BC nanotherapy

The successful translation of nanoscale therapies for breast cancer treatment hinges not only on their intrinsic properties but also on their interactions with biological systems, notably the formation of a protein corona upon exposure to biological fluids. The protein corona, composed of biomolecules such as proteins, lipids, and other biomolecules, plays a pivotal role in determining the fate and functionality of nanoparticles within the body. Recent advancements have highlighted the significance of personalized protein coronas in modulating the safety, efficacy, and therapeutic outcomes of nanoscale materials in cancer therapy.^201^

### 8.1. Formation and composition of personalized protein corona

Upon introduction into physiological environments, nanoparticles rapidly adsorb proteins from the surrounding milieu, forming a dynamic protein corona layer. The composition of this corona is highly variable and influenced by factors such as nanoparticle surface properties, size, shape, and surface charge, as well as the unique biological identity of the individual. Recent studies have revealed substantial inter-individual variability in protein corona composition, emphasizing the need for personalized approaches in nanomedicine.^202^

### 8.2. Impact on nanoparticle–biological interactions

The protein corona serves as a molecular interface between nanoparticles and biological entities, influencing their interactions with cells, tissues, and physiological barriers. In the context of breast cancer, the protein corona can modulate nanoparticle uptake by cancer cells, intracellular trafficking, and subsequent therapeutic responses. Additionally, the protein corona may affect nanoparticle pharmacokinetics, biodistribution, and clearance profiles, thereby shaping their overall therapeutic efficacy and safety profiles.^203^

### 8.3. Implications for precision medicine in breast cancer treatment

The recognition of personalized protein coronas as critical determinants of nanoscale therapy outcomes underscores the importance of integrating precision medicine approaches into the development and optimization of nanoparticle-based breast cancer treatments. By accounting for individual variations in protein corona composition, it may be possible to tailor nanotherapeutic strategies to optimize treatment responses and minimize adverse effects in breast cancer patients.^204^

### 8.4. Challenges and future directions

Despite significant progress in understanding the role of personalized protein coronas in nanoscale therapies, several challenges remain. These include the development of robust predictive models for protein corona formation, strategies to mitigate inter-individual variability, and the translation of personalized nanomedicine approaches into clinical practice. Future research efforts should focus on addressing these challenges to realize the full potential of personalized protein corona-based approaches in breast cancer treatment.^205^

## 9. Conclusions and future prospects

**Fig. 7:**
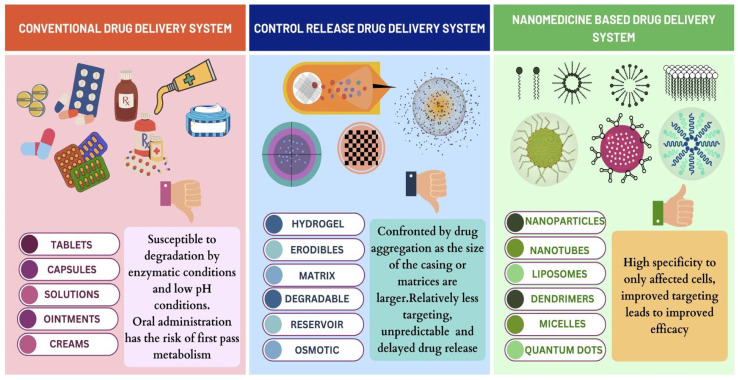
Comparison of drug delivery in breast cancer.

BC chemotherapy in the form of novel nanotechnology will continue to benefit amidst the increasing challenges faced in tumor treatment. Nanomedicine has a wide range of advantages for cancer patients. It is important to note that the inclusion of nanomaterials results in better stability, improved solubility and controlled release kinetics for chemotherapeutic agents. Nanoformulations have the potential to overcome the significant limitations of conventional BC therapy, as summarized in Table 4. This review clearly describes the various drug delivery systems established for BC treatment and highlights the importance of nanotherapy (Fig. 7). However, the complicated aspects of nanomedicine use have yet to be fully resolved, and additional research is needed. Scientists are constantly working to develop new nanoformulations that require adequate evidence that nanoformulations are therapeutically much better, abundantly stable and rational.

**Table 4:** Summary of challenges in standard breast cancer treatment and how nanomedicine can help to overcome these challenges

Limitations of standard breast cancer therapy	Highlights of nanotechnology in BC chemotherapy	Reference
Lack of selectivity in drug targeting for BC	Both passive as well as active targeting increases drug concentration at tumor site and diminishes toxic drug levels in noncancer sites	189
Improper reach of drugs to metastatic organs including brain, bone and lungs	Nanotherapy has in-born features of brain and bone penetration	190
Unfavorable PK properties like short half-life and rapid clearance	Approaches like PEGylation can be applied to extend the retention of the drug	191
Influence of the drugs or excipients leading to dose-limiting toxicity, say, surfactants or organic cosolvents	Solvent-free, surfactant-free nanoformulations can be formulated for controlled-release of drugs	192
Cellular level drug resistance, for instance, increase in efflux transport of drug	Endocytosis takes place as a result of passive/active transport; some nano preprations inhibit efflux mechanism and codelivery of agents that promote drug resistance	193
TME level drug resistance like low pH, hypoxia, *etc.*	Possibility of targeting TME and use of stimulus-sensitive approaches	194
Challenges in destroying cancer stem cells	Cancer stem cells can be directly targeted	195
Inefficient pharmaceutical characteristics of the drugs such as low water-solubility, poor *in vivo* stability	Drug solubilization can be achieved easily by means of nanotechnology and can even protect the unstable drugs	196
Suboptimal dosing schedule, particularly in case of using multiple drugs as combinations	Dosing schedule can be consciously optimized and delivery of multiple drugs is made better	197

## Abbreviations

ABC: Accelerated blood clearance
API: Active pharmaceutical ingredient
AUC: Area under the curve
BA: Bioavailability
BC: Breast cancer
BCSC: Breast cancer stem cells
BTZ: Bortezomib
CDK: Cyclin-dependent kinase
CDs: Carbon dots
Cmax: Peak free drug concentration
CNT: Carbon nanotubes
DDS: Drug delivery system
DEX: Dextran
DMPC: Dipalmitoylphosphatidylcholine
DOPE: Dioleoyl phosphoethanolamine
DOX: Doxorubicin
DPPC: Dipalmitoyl phosphatidylcholine
DSPC: Distearoyl phosphatidylcholine
EPR: Enhanced permeability and retention
ER: Estrogen receptor
FA: Folic acid
Gd: Gadolinium
GEM: Gemcitabine
GI: Gastro intestinal
GPI: Glyco phosphatidyl inositol
GSTE: Gloriosa superba tuber extract
HeLa: Human cervical carcinoma
HER2/ERBB2/EGFR: Human epidermal growth factor receptor/oncogene 2
HSA: Human serum albumin
i.v: Intravenous
IgG: Immunoglobulin G
LA: Lauric acid
LNP: Liposomal nanoparticles
MAb: Monoclonnal antibody
MRI: Magnetic resonance imaging
MSN: Mesoporous silica nanoparticles
MV: Microvesicles
nab: Nanoparticle-albumin bound
neu: Neutrophil
NLC: Nanostructured lipid carriers
NP: Nanoparticle
NSCLC: Non-small-cell lung carcinoma
PAA: Poly acrylic acid
PAMAM: Polyamidoamine
PARP: Poly ADP ribose polymerase
PEG: Poly ethylene glycol
PEO: Polyethylene oxide
PFOA: Perfluorooctanesulfonic acid
PLA: Poly lactic acid
PLGA: Poly d,l-lactic-*co*-glycolic acid
PNP: Polymeric nanoparticle
PPI: Propylene imine
PR: Progesterone receptor
PTX: Paclitaxel
PVX: Potato virus X
QDs: Quantum dots
RES: Reticuloendothelial system
ROS: Reactive oxygen species
SLN: Solid lipid nanoparticles
SMEDDS: Self-micro emulsifying drug delivery system
SNEDDS: Self-nano emulsifying drug delivery system
SPIONs: Super paramagnetic iron oxide nanoparticles
SWNT/MWNT: Single-walled/multi-walled carbon nanotubes
TCDD: 2,3,7,8-Tetrachlorodibenzo-*p*-dioxin
Tmax: Time taken to reach peak free drug concentration
TME: Tumor microenvironment
TNBC: Triple negative breast cancer
TSL: Thermo-sensitive liposomes
USFDA: United States food and drug administration
WHO: World health organization

## Author contributions

Sruthi Laakshmi Mugundhan designed the graphical illustrations and contributed to the writing, conceptualization and publishing process; Mothilal Mohan contributed to the supervision, drafting of the work, revision of the intellectual content and final approval of the manuscript. All the authors read and approved the final manuscript.

## Conflicts of interest

There are no conflicts to declare.

## Supplementary Material
